# Built Environment: New Default for Asphalt?

**DOI:** 10.1289/ehp.116-a379a

**Published:** 2008-09

**Authors:** Lance Frazer

The vast majority of the nearly 2 million miles of paved roads in the United States are surfaced with asphalt pavement, which is made by combining a thick hydrocarbon mixture known as liquid asphalt binder with sand, gravel, or crushed stone (“aggregate”). Each year about 60 million tons of hot-mix asphalt (HMA) pavement are laid on U.S. roads, according to figures presented last spring at the 12th Annual Minnesota Pavement Conference. Asphalt pavement is tough, flexible, and easy to repair, but the commonly used HMA is energy-intensive to produce, releases greenhouse gases, and poses potential hazards for workers. So researchers are looking at lower-temperature asphalt pavements as a way around these problems.

Warm-mix asphalt (WMA) can be used at temperatures of 212–284°F, about 50–100°F cooler than HMA, while cold-mix asphalt (CMA) is used at ambient temperatures. Both can be produced with minimal modifications to HMA plants, says University of Wisconsin–Madison civil and environmental engineering professor Hussain Bahia.

In paving workers, inhalation of asphalt fumes can irritate the nose, throat, and lungs, as well as cause excessive fatigue and loss of appetite, according to the National Institute for Occupational Safety and Health. A study in the November 2004 issue of the *Annals of Occupational Hygiene* cited dermal and inhalational exposure of paving workers to polycyclic aromatic compounds, which have been labeled as reasonably anticipated to be human carcinogens by the National Toxicology Program. But whereas certain extracts of asphalt have caused a carcinogenic skin response in experimental animals, research to date has found no conclusive evidence of increased risk of skin or lung cancer in workers.

HMA plants use petrofuels to heat the liquid asphalt binder to a workable temperature as well as dry and heat the aggregate to improve cohesion. “You inevitably have . . . the resultant emission of typical fuel combustion by-products like sulfur dioxide, carbon monoxide, carbon dioxide, volatile organics, and other substances, similar to a home heating furnace,” says Gary Fore, vice president for environment, health, and safety at the National Asphalt Pavement Association (NAPA), a trade association. “That’s why we’re continuing to explore lower-temperature alternatives.”

In the September 2007 *Europeanroads Review*, Pierre Dorchies and colleagues wrote that CMA technology could afford a 30% energy saving over traditional HMA. NAPA president Mike Acott says, “The challenge with cold mix is to produce a surface as strong and reliable as hot mix, and there are some factors getting in the way. Cold mix is not generally used as a surfacing material and certainly not for roads subjected to medium to heavy traffic.” CMA is used in countries such as South Africa and India, where there is relatively little heavy traffic, and to a lesser degree in the United States.

The issue, Acott says, “is to produce materials that perform as well as hot mix for the U.S. road infrastructure. That’s where we believe warm mix comes in. Our research indicates that warm mix can produce a surface laid down at a substantially lower temperature [that performs] as well as hot mix.”

The 2007 NAPA report *Warm-Mix Asphalt: Best Practices* says a shift from HMA to WMA in Norway, Italy, the Netherlands, France, and Canada has yielded significant emissions reductions. Adoption of WMA in these nations is being driven largely by Europe’s participation in the Kyoto Protocol and implementation of the new Registration, Evaluation, Authorisation and Restriction of Chemical Substances (REACH) legislation, according to an article in the 14 April 2008 issue of *Michigan Contractor and Builder*. WMA shows signs of being as good as or better than HMA, but with a track record of only about 10 years, it hasn’t yet had time to prove itself in real-world settings. More data should be forthcoming: in 2008 the Asphalt Institute was awarded a $900 million 3-year grant by the Transportation Research Board of the National Academies to compare elements of WMA and HMA including performance and emissions.

Peter Grass, president of the Asphalt Institute, another trade association, says several projects using WMA are under way in the United States, including plans this year to lay more than 1 million tons of WMA in Texas alone. The Massachusetts Port Authority Board has also just authorized $6.3 million to repave a runway at Boston Logan International Airport with WMA, making Logan the first U.S. airport to use the more environmentally friendly surface. In a 24 July 2008 press release, Port Authority Board CEO and executive director Thomas J. Kinton Jr. said, “Warm mix uses 20% less energy to make, produces 20% fewer greenhouse emissions when applied, and allows us to use a higher percentage of recycled asphalt pavement in the final product.”

There is still a lot of research to be done before all the questions are answered and issues settled about CMA, a point with which Bahia agrees. Within his newly established Modified Asphalt Research Center, Bahia is exploring what it will take to make CMA a recognized replacement for HMA, including the possible addition of polymers or plastics to yield a quieter, safer, more durable pavement. Bahia says “Some 80% of the roads in this country are low-traffic-volume roads. Those are the applications where we believe cold mix would be appropriate. As energy prices continue to rise, taking asphalt prices along, we’re going to be forced to consider alternatives.”

## Figures and Tables

**Figure:**
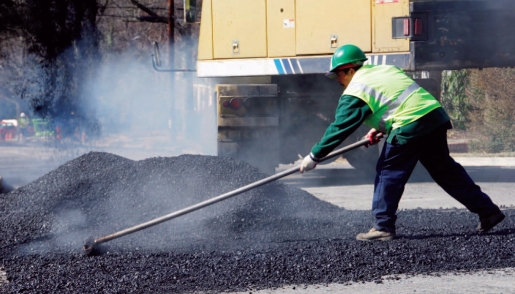
Cooler asphalt offers one route to more sustainable road building

